# NUAK2 and RCan2 participate in the p53 mutant pro-tumorigenic network

**DOI:** 10.1186/s13062-021-00296-5

**Published:** 2021-08-04

**Authors:** Eleonora Mammarella, Carlotta Zampieri, Emanuele Panatta, Gerry Melino, Ivano Amelio

**Affiliations:** 1 Department of Experimental Medicine, TOR, University of Rome Tor Vergata, 00133 Rome, Italy; 2 School of Life Sciences, University of Nottingham, Nottingham, UK

**Keywords:** Tumour suppression, Metastasis, Tumour progression, Cancer prognosis

## Abstract

Most inactivating mutations in TP53 gene generates neomorphic forms of p53 proteins that experimental evidence and clinical observations suggest to exert gain-of-function effects. While massive effort has been deployed in the dissection of wild type p53 transcriptional programme, p53 mutant pro-tumorigenic gene network is still largely elusive. To help dissecting the molecular basis of p53 mutant GOF, we performed an analysis of a fully annotated genomic and transcriptomic human pancreatic adenocarcinoma to select candidate players of p53 mutant network on the basis their differential expression between p53 mutant and p53 wild-type cohorts and their prognostic value. We identified NUAK2 and RCan2 whose p53 mutant GOF-dependent regulation was further validated in pancreatic cancer cellular model. Our data demonstrated that p53^R270H^ can physically bind RCan2 gene locus in regulatory regions corresponding to the chromatin permissive areas where known binding partners of p53 mutant, such as p63 and Srebp, bind. Overall, starting from clinically relevant data and progressing into experimental validation, our work suggests NUAK2 and RCan2 as novel candidate players of the p53 mutant pro-tumorigenic network whose prognostic and therapeutic interest might attract future studies.

## Introduction

Structural lesions or functional impairment in the TP53 gene are among the most common genetic events in human cancers. Typically occurring as missense mutations (over 80% of the cases), they involve the DNA binding domain either in DNA contact residues or in residues that have important implication for the conformational structure of p53 [1–4]; thus, p53 missense mutations generally result in generation of p53 mutant proteins. Evidence from genetically engineered mouse models indicates that the presence of p53 mutant forms facilitates development of more aggressive and metastatic tumours compared to those arising in p53^−\−^ mice [5–10]. Moreover, mouse models with inactivatable p53 hotspot mutation demonstrated that tumours depend on sustained mutant p53 expression [11–14]. Hence, substantial experimental evidence supports the existence of a gain-of-function (GOF) activities independent of their effects on wild-type p53.

With the massive effort of genomic studies and precision oncology approaches [15–20], it is well understood that millions of patients worldwide live with a p53 mutant expressing tumour, with subsequent potential defects in cell death [21–24] or autophagy [25–29], however this information does not currently reflect a benefit for patients as effective therapeutic approaches to target p53 gain-of-function (GOF) are still lacking. As p53 mutant proteins appear to be generally undruggable, deconvolution of the gene network mediating its oncogenic effect is proposed as a promising strategy to improve anti-cancer therapies and to complement the substantial effort of defining the wt p53 tumour suppressive network [30–33]. Mutations of TP53 genes has been linked with a causative relationship with genetic progression of pancreatic adenocarcinoma (PDAC) [34]. PDAC emerges from an original indolent pancreatic intraepithelial lesion (PanINs) that persist in a poorly aggressive forms for many years. Progression of PanINs to highly aggressive, frankly invasive, and metastatic PDACs is very frequently associated to p53 mutations (75% of cases) [35–37] and directs a complex rearrangement of the microenvironment [38–40]. This latter staged neoplastic forms become symptomatic, but even when surgically approachable they generally manifest too late to carry positive prognosis. Specific GOF mechanisms have been ascribed to p53 mutant in PDAC progression, including the deregulation of the other p53 family member p73 [41–43] in a molecular axis involving the transcriptional factor NF-Y [44]. Conversely, the other p53 family member, p63 [45–48], although considered a master regulator of pancreatic cancer squamous lineage specification [49, 50] and frequently associated to p53 GOF in other models [51, 52], has not yet emerged with a causative link in driving p53 GOF phenotype of PDAC.

While mutant p53 has emerged to direct metastatic programme in PDAC mouse models, the dissection of the gene network has not yet provided promising druggable targets. Here, we attempt to improve our understanding of the gene transcriptional network regulated by mutant p53 in the pathogenesis of PDAC. Based on their prognostic impact we stratified the genes mostly differentially expressed in human PDAC carrying mutant p53 versus wt p53 and we identified NUAK2 and the RCan2 (regulator of calcineurin-2) as potential mediators of p53 mutant pro-tumorigenic network. Using a model of pancreatic adenocarcinoma cell line derived from *pdx1-CR*E mouse models with pancreas-specific expression of oncogenic KRAS (*LSL-KRAS*^*G12D*^) and p53^R270H^ mutation (homologue of human R273H) we confirmed NUAK2 and RCan2 regulation and identified the potential underlying molecular mechanisms responsible for their p53^R270H^-mediated gene expression. Our study suggests a potential significance of mutant p53/NUAK2-RCan2 axis that might direct future studies in this area of research.

## Materials and methods

### Cell culture and transfection

Mouse pancreatic cancer cell lines (KPC270) [6] were cultured in DMEM medium (Gibco) supplemented with 10% fetal bovine serum (FBS, Gibco) and penicillin/streptomycin (2 units/ml) (Gibco) at 37 °C under 5% CO_2_, as previously described [53, 54]. siRNA transfection was carried out using Lipofectamine RNAiMAX (Invitrogen) with 50 nM Silencer Select Pre-designed trp53 (Ambion, siRNA ID s75472), RCan2 (Ambion siRNA ID: s203908) and Silencer Select Negative Control No. 1 siRNA (Ambion).

### Chromatin immunoprecipitation (ChIP)

To perform ChIP assays, 1% formaldehyde for 10 min was used to cross-link the proteins to the DNA, then the reaction was quenched with 0.125 M glycine. After nuclei lysis, the lysates were sonicated and the immunoprecipitation was carried out using Dynabeads Protein G (Invitrogen, cat. 10004D). 0,1 mg/mL RNase A (Thermo Scientific) and Proteinase K (20 mg/mL, Thermo Scientific) were used to reverse the cross-links. The DNA was purified by QIAquick PCR kit (QIAGEN). The DNA levels were measured by real-time quantitative PCR. ChIP was performed with the following antibodies: anti-p53 (Leica, cat. P53-CM5P-L), and mouse IgG Isotype control (Invitrogen, cat. 10500C).

### RNA extraction, reverse transcription and real-time qPCR analysis.

RNA was isolated from cells using RNeasy Mini Kit (Qiagen) according to the manufacturers’ protocols [55, 56]. The concentration and purity were detected by Nanodrop. RNA was reverse-transcribed into cDNA using with SensiFAST cDNA Synthesis Kit (Meridian Bioscience, BIO-65054). The relative RNA expression levels were determined by real-time PCR with Fast SYBR Green PCR Master Mix (Applied Biosystems). Results were calculated using TBP mRNA as a normalizer.

### Live cell imaging analysis

Live cell imaging was performed by using IncuCyte® Live-Cell Analysis Systems, treating the cells. 24 h after transfection, the cells were seeded into a 96-well and placed into the Incucyte® Live-Cell Analysis System to monitor phase contrast every 3 h. Scratch was performed with appropriate equipment provided by Sartorius. The analysis was performed using Incucyte Basic Analysis Software for proliferation and Incucyte Scratch Wound Analysis for the migration.

### Bioinformatics analyses

The human pancreatic adenocarcinoma data were obtained from PanCancer Atlas TCGA dataset study. For Kaplan–Meier survival analysis, the entire patient cohort was divided into two groups depending on the p53 status (mutated, not mutated).

The Chip-Seq database analysis was executed through Chip Atlas (http://chip-atlas.org/peak_browser) and Integrative Genomics Viewer (http://www.broadinstitute.org/igv/) for peaks visualizing. From Chip-Atlas we designed primers for Chip Assay on Rcan2 gene based on peaks binding for Srebf1 (Chip-Seq id = SRX1650053) and trp63 (Chip-Seq id = SRX3205488) using UCSC Genome Browser (http://genome.ucsc.edu/index.html).

### Statistics

Statistical analysis was performed using GraphPad Prism 9.0 (GraphPad Software Inc.). All results are expressed as the mean ± SEM. RT-qPCR data were analyzed by t-test (*p < 0.05, **p < 0.01, ***p < 0.001). The Kaplan–Meier method and Mantel-Cox test were applied to determine the progress-free survivals and overall survivals between different patients. All the experiments were performed at least three biological repeats.

## Results and discussion

Cancer genomic studies have seen an exponential growth in the last decade [57–60], and a massive amount of data has now become openly accessible. Analyses of this information can effectively direct studies addressing the activation of specific gene network and their prognostic significance in cancer pathogenesis [61–63]. We reasoned therefore that identification of potentially clinically and biologically relevant molecular network mediated by p53 mutant might have been directed by the analyses of a large cancer patients’ dataset, fully annotated with clinical and molecular information. To this end, we selected the PDAC cohort from the TCGA PanCancer Atlas [64], which included full genomic information (mutations, structural variations, and putative copy number alterations), in addition to full transcriptional profiling (RNA-seq), protein expression and clinical variables. To obtain potential clinically relevant data, we decided to select genes of interest on the basis (1) of statistically significant differential expression between p53 mutant and p53 wild-type cohorts and (2) of their prognostic significance assessed as measure of patients’ survival prediction.

**Fig. 1:**
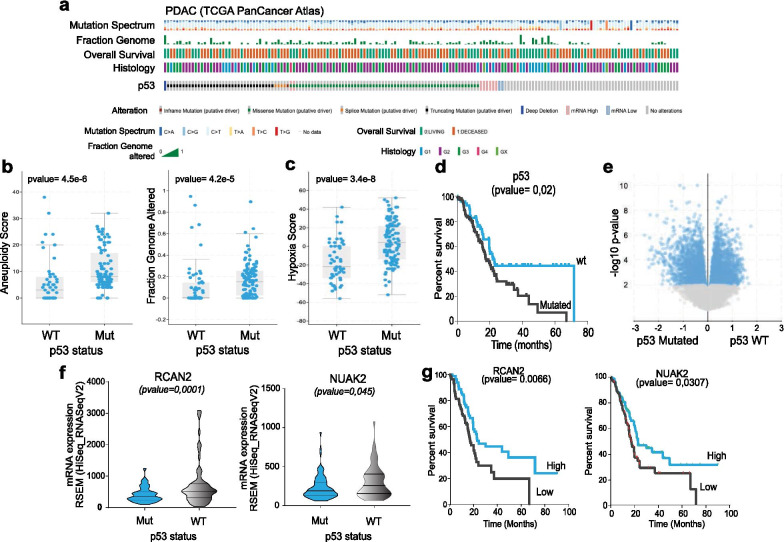
RCan2 and NUAK2 emerge as part of mutant p53 network in a human PDAC cohort. **a** Oncoprint diagram reports mutation spectrum, fraction genome, overall survival, histology and p53 mutational status in the PDAC cohort from the TCGA PanCancer Atlas. **b**,** c** Samples from p53 mutant patients display higher “aneuploidy score”, “genome altered” and “hypoxia score” if compared to the p53 wt cohort. **d** Kaplan–Meier plot reports overall survival of p53 mutant vs p53 wt PDAC patients from the TCGA PanCancer Atlas. **e** Volcano plot reports genes differentially regulated between p53 mutant versus p53 wt PDAC patients from the TCGA PanCancer Atlas. **f** mRNA expression level (from RNA-seq) data of RCAN2 and NUAK2 display differential expression between p53 mutant versus p53 wt PDAC patients (TCGA PanCancer Atlas). **g** Kaplan–Meier plot reports overall survival of patients with RCan2 and NUAK2 highly expressing PDAC (TCGA PanCancer Atlas). *Source*: cbioportal.org

The PDAC cohort in the TCGA PanCancer Atlas includes 184 cases, 66% of which display p53 mutation; these have a significant prevalence of missense mutations, but also include a significant fraction of truncation (Fig. 1a). At the genomic level, p53 inactivation was significantly associated to hallmarks of genomic instability, assessed as “aneuploidy score” and “genome altered” (Fig. 1b). This data confirms that p53 inactivation well defines a subset of tumour with high genetic plasticity [65, 66], in addition to alternative mechanism of therapy resistance and metastasis [67]. Moreover, in keeping with our previous findings, p53 mutants also correlated with “hypoxia score” (Fig. 1c), indicating that the GOF mutant impinges in the hypoxia transcriptional response and supporting of postulation of a context (hypoxia) dependent p53 GOF effect [68–70]. We queried this dataset for transcriptomic data (RNA-seq) searching for the significantly differentially expressed genes in the p53 mutant and p53 wild type cohorts. 2171 genes were enriched in the p53 mutant cohort, while 2433 were correlated to the p53 wt group, which displayed a better prognosis (Fig. 1d, e). Next, we stratified these for their prognostic value, selecting the genes which were displaying a predictive effect on patients’ survival consistent with the prognostic value shown by p53 inactivation. From this analysis we selected two putative genes, namely NUAK2 and RCan2, whose participation in the p53 mutant pro-tumorigenic network have never been explored (Fig. 1f, g).

**Fig. 2:**
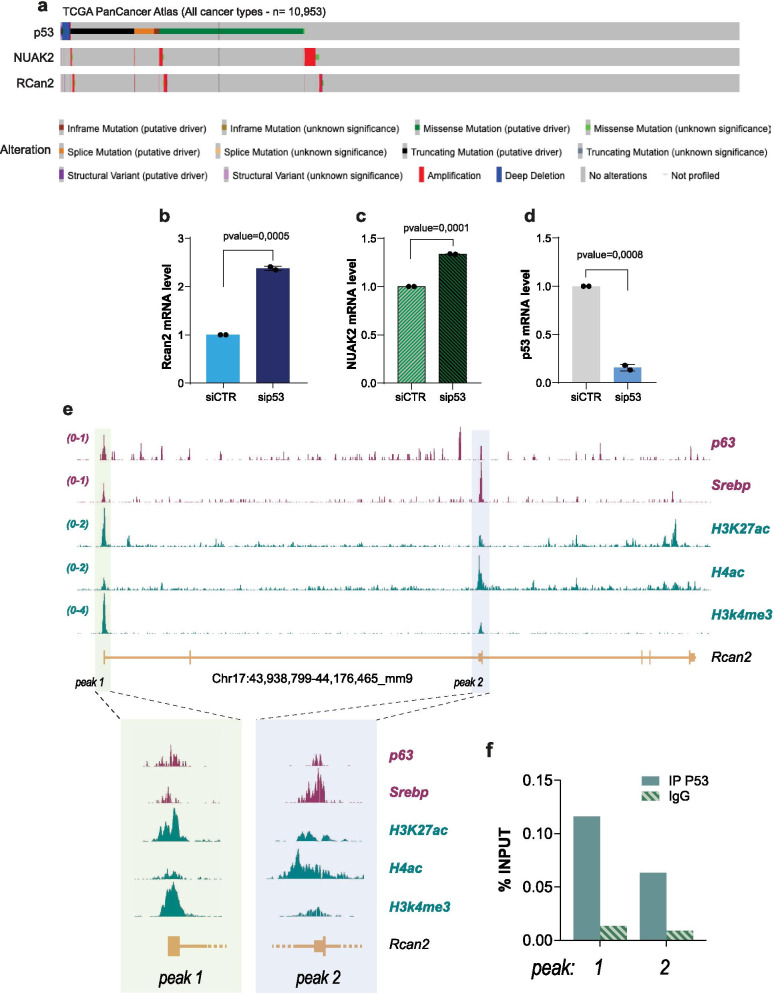
Mutant p53 regulates a RCan2 expression via GOF effects. **a** Oncoprint diagram reports mutational status of RCan2 and NUAK2 in full cohort of TCGA PanCancer Atlas. **b**–**d** mRNA level of RCan2, NUAK2 and p53 in KPC^270^ cells following p53 silencing. **e** ChIP-seq profile of histone post-translational modifications, transcriptional factors binding in the genomic region of mouse RCan2. **f** ChIP-qPCR for p53 binding on peak 1 and peak 2 in KPC^270^ cells

NUAK2 is a serine/threonine kinase, belonging to the family of AMPK, whose mutations are associated to congenital severe neurodevelopmental defects [71], although it is rarely observed mutated in cancer (Fig. 2a). NUAK2 activity has been implicated in YAP-driven growth and deregulation of this axis might have significance for progression and therapy of hepathocarcinoma [72] and other cancer types [73]. RCan2 is an inhibitor of calcineurin and therefore influences the calcineurin-nuclear factor of activated T cells (NFAT) signaling, and downstream biological consequences include differentiation/proliferation [74, 75], metabolism and oxidative stress response [76–79]. RCan2 mutations are also rarely observed in human cancers (Fig. 2a), but KRas mutations lead to repression of RCan2 in colorectal cancer mouse models, influencing NFAT-dependent regulation of cancer cell proliferation [80]. So far, no direct physical or functional interaction has been demonstrated for these two genes. Nonetheless, a potential p53 mutant-dependent regulation of these two genes might have direct implication in the biology of cancer cells.

With the analysis of clinical data (Fig. 1) we identified a reduced expression of both NUAK2 and RCan2 in the p53 mutant cohort compared to the wt cohort. Despite these data associate both genes to p53 mutations, the analysis per se does not directly imply a regulation mediated by a GOF mechanism, as this result might purely reflect a positive regulation exerted by wt p53 (i.e., upregulation in the p53 wt cohort). To discriminate between these two possibilities, we employed a mouse cellular model of p53 mutant PDAC. We selected a pancreatic adenocarcinoma cell line derived from *pdx1-CR*E mouse model with pancreas-specific expression of oncogenic KRAS (*LSL-KRAS*^*G12D*^) and p53^R270H^ mutation (homologue of human R273H), hereafter referred as KPC^270^ cells. p53^R270H^ mutation is within the most frequently observed missense mutations in all cancer types and in particular in PDAC. Depletion of p53^R270H^ by siRNA-mediated silencing in KPC^270^ cells led to a significant upregulation of NUAK2 and RCan2 (Fig. 2b–d). The direction of the regulation of both genes appeared consistent with the results of the clinical data, and importantly supported a direct implication of p53^R270H^-dependent GOF effect. Remarkably however, while upregulation of RCan2 mRNA was reaching a substantial 2.5-fold increase, the alteration of NUAK2 mRNA level appeared marginal (1.3-fold increase) to justify at least in this cellular experimental model a significant biological relevance. Hence, we decided to carry on the study of RCan2 regulation.

To further investigate the basis of p53^R270H^/RCan2 axis and define the molecular underlying mechanism of the GOF effect, we explored ChIP-seq data to identify potential regulatory regions in RCan2 gene locus that might be susceptible to p53^R270H^. The mechanisms of p53 mutant regulation of gene expression have been frequently ascribed to its ability to interact with transcriptional factors and influence their activity on gene regulatory genomic regions. The activity of the transcriptional factors p63, HIF-1, NY-F and Srebp was shown to be influenced by p53 mutations, hence we searched for binding enrichment in RCan2 gene locus of known p53 mutant protein partners querying ChIP-seq datasets. We identified binding of p63 and Srebp in two specific regions of mouse RCan2, hereafter referred as peak 1 and peak 2 (Fig. 2e). Remarkably, the binding of p63 and Srebp appeared to largely overlap in the same genomic regions, that importantly appeared to be also enriched for permissive histone posttranslational modifications [81], such as acetylation of lysine 27 of histone 3 (H3K27ac), trimethylation of lysine 4 of histone 3 (H3K4me3) and acetylation of histone 4 (H4ac) (Fig. 2e). Hence overall, these two genomic areas appeared active regulatory regions that might potentially be sensitive to mutant p53. To test the hypothesis that mutant p53 executes a GOF effect on RCan2 by physically binding its genomic locus, we performed ChIP-qPCR analysis in KPC^270^ cells for p53 binding in peak 1 and peak 2. qPCR analysis confirmed a specific and selective binding of p53^R270H^ in both regions (Fig. 2f). This set of data demonstrated a GOF mechanism mediating a direct transcriptional regulation by p53^R270H^ on RCan2 gene expression, that might involve p63 and Srebp.

**Fig. 3:**
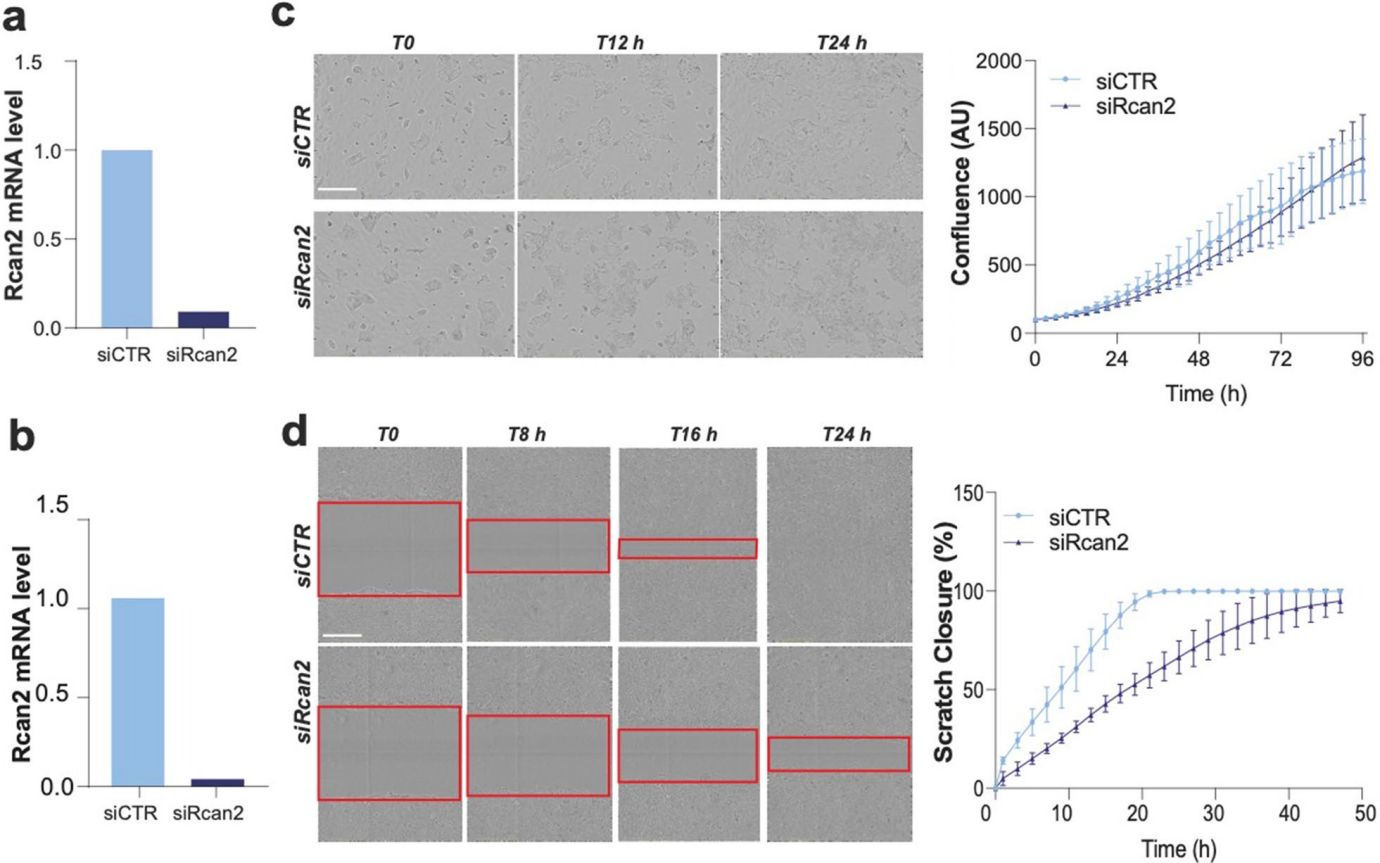
RCan2 expression influences cell motility but not proliferation. **a**,** b** p53 mRNA level in KPC^270^ cells following p53 silencing. **c**,** d** In vivo live cell imaging analysis by IncuCyte platform measured cell growth (phase contrast) in **c** and migration (scratch closure measured as scratch area by phase contrast) in **d** in KPC^270^ cells following RCan2 silencing. Red boxes indicate the scratch area. Scale bars 400 microns

Next, we asked that biological consequences of RCan2 regulation on the pro-tumorigenic properties of KPC^270^ cells. To address this point, we employed live cell imaging techniques (IncuCyte technology) following modulation of RCan2 expression to measure cell proliferation and migration of KPC^270^ cells, as in vitro readout of cancer cell biology. Depletion of RCan2 was effectively achieved in KPC^270^ cells by siRNA silencing (Fig. 3a, b). Time-lapse analysis of siCTR and siRCan2 transfected cells displayed no significant alteration in the proliferation capacity of KPC^270^ cells over a timeframe of 96 h (Fig. 3c). On contrary the migration capacity was substantially reduced following siRCan2 transfection, with control cells reaching 100% of “scratch closure” in approximately 24 h compared to 48 h required to the RCan2-depleted cells (Fig. 3d). While the regulation mediated by RCan2 on cell motility might implicate this gene in the process of metastasis, it would contradict the prognostic impact and the reverse correlation with p53 mutant (Figs. 1 and 2). Therefore, the alterations of the migratory capacity of KPC^270^ cells appears not associate to the significance of the p53^R270H^/RCan2 molecular axis and will therefore require further investigation to understand the biological relevance and the appropriate biological context.

Overall, this work expands our understanding of the mutant p53 pro-tumorigenic gene network, providing novel effectors whose biological significance and therapeutic interest might be explored in the future, especially in the light of the development of precision oncology [82–86]. The interest of our data lies in the clinical analysis that supports a possible relevance of the findings. NUAK2 and RCan2 were identified as genes differentially regulated with a consistent prognostic value in human PDAC carrying p53 mutations. Our cellular experimental model allowed to validate the correlative nature of the clinical data as a molecular regulation mediated by p53^R270H^ GOF effect, implicating genomic regulatory elements at the mechanistic level in the process. While further work will help determining the importance of p53^R270H^/RCan2-NUAK2 in PDAC pathogenesis, our study represents a proof-of-principle approach for dissection of the mutant p53 pro-tumorigenic network and identification of novel therapeutic targets and prognostic factors in cancer pathogenies.

## Data Availability

Available upon requests.

## References

[1] Halazonetis TD, Kandil AN (1993). Conformational shifts propagate from the oligomerization domain of p53 to its tetrameric DNA binding domain and restore DNA binding to select p53 mutants. EMBO J.

[2] Wright JD, Lim C (2007). Mechanism of DNA-binding loss upon single-point mutation in p53. J Biosci.

[3] Wieczorek AM, Waterman JL, Waterman MJ, Halazonetis TD (1996). Structure-based rescue of common tumor-derived p53 mutants. Nat Med.

[4] Ham SW, Jeon HY, Jin X, Kim EJ, Kim JK, Shin YJ, Lee Y, Kim SH, Lee SY, Seo S (2019). TP53 gain-of-function mutation promotes inflammation in glioblastoma. Cell Death Differ.

[5] Lang GA, Iwakuma T, Suh YA, Liu G, Rao VA, Parant JM, Valentin-Vega YA, Terzian T, Caldwell LC, Strong LC (2004). Gain of function of a p53 hot spot mutation in a mouse model of Li-Fraumeni syndrome. Cell.

[6] Olive KP, Tuveson DA, Ruhe ZC, Yin B, Willis NA, Bronson RT, Crowley D, Jacks T (2004). Mutant p53 gain of function in two mouse models of Li-Fraumeni syndrome. Cell.

[7] Donehower LA, Lozano G (2009). 20 years studying p53 functions in genetically engineered mice. Nat Rev Cancer.

[8] Kadosh E, Snir-Alkalay I, Venkatachalam A, May S, Lasry A, Elyada E, Zinger A, Shaham M, Vaalani G, Mernberger M (2020). The gut microbiome switches mutant p53 from tumour-suppressive to oncogenic. Nature.

[9] Mantovani F, Collavin L, Del Sal G (2019). Mutant p53 as a guardian of the cancer cell. Cell Death Differ.

[10] Fedorova O, Daks A, Shuvalov O, Kizenko A, Petukhov A, Gnennaya Y, Barlev N (2020). Attenuation of p53 mutant as an approach for treatment Her2-positive cancer. Cell Death Discov.

[11] Alexandrova EM, Yallowitz AR, Li D, Xu S, Schulz R, Proia DA, Lozano G, Dobbelstein M, Moll UM (2015). Improving survival by exploiting tumour dependence on stabilized mutant p53 for treatment. Nature.

[12] Schulz-Heddergott R, Stark N, Edmunds SJ, Li J, Conradi LC, Bohnenberger H, Ceteci F, Greten FR, Dobbelstein M, Moll UM (2018). Therapeutic ablation of gain-of-function mutant p53 in colorectal cancer inhibits Stat3-mediated tumor growth and invasion. Cancer Cell.

[13] Lonetto G, Koifman G, Silberman A, Attery A, Solomon H, Levin-Zaidman S, Goldfinger N, Porat Z, Erez A, Rotter V (2019). Mutant p53-dependent mitochondrial metabolic alterations in a mesenchymal stem cell-based model of progressive malignancy. Cell Death Differ.

[14] Celardo I, Melino G, Amelio I (2020). Commensal microbes and p53 in cancer progression. Biol Direct.

[15] Amelio I, Bertolo R, Bove P, Buonomo OC, Candi E, Chiocchi M, Cipriani C, Di Daniele N, Ganini C, Juhl H (2020). Liquid biopsies and cancer omics. Cell Death Discov.

[16] Ranganathan P, Chinnaswamy G, Sengar M, Gadgil D, Thiagarajan S, Bhargava B, Booth CM, Buyse M, Chopra S, Frampton C, et al. The International Collaboration for Research methods Development in Oncology (CReDO) workshops: shaping the future of global oncology research. Lancet Oncol. 2021. 10.1016/S1470-2045(21)00077-2 PMC8328959 34216541

[17] Panchin AY, Aleoshin VV, Panchin YV (2019). From tumors to species: a SCANDAL hypothesis. Biol Direct.

[18] Khairi S, Osborne J, Jacobs MF, Clines GT, Miller BS, Hughes DT, Else T (2020). Outcome of clinical genetic testing in patients with features suggestive for hereditary predisposition to PTH-mediated hypercalcemia. Horm Cancer.

[19] Pekic S, Soldatovic I, Miljic D, Stojanovic M, Doknic M, Petakov M, Popovic V (2019). Familial cancer clustering in patients with prolactinoma. Horm Cancer.

[20] Neuzillet Y, Raynaud JP, Dreyfus JF, Radulescu C, Rouanne M, Schneider M, Krish S, Roupret M, Drouin SJ, Comperat E (2019). Aggressiveness of localized prostate cancer: the key value of testosterone deficiency evaluated by both total and bioavailable testosterone: AndroCan study results. Horm Cancer.

[21] Amaral MP, Branco LM, Strasser A, Dixit VM, Bortoluci KR (2020). Paradise revealed III: why so many ways to die? Apoptosis, necroptosis, pyroptosis, and beyond. Cell Death Differ.

[22] Liu Y, Leslie PL, Zhang Y (2021). Life and death decision-making by p53 and implications for cancer immunotherapy. Trends Cancer.

[23] Senichkin VV, Streletskaia AY, Gorbunova AS, Zhivotovsky B, Kopeina GS (2020). Saga of Mcl-1: regulation from transcription to degradation. Cell Death Differ.

[24] Krenning L, van den Berg J, Medema RH (2019). Life or death after a break: what determines the choice?. Mol Cell.

[25] Shi Y, Norberg E, Vakifahmetoglu-Norberg H (2020). Mutant p53 as a regulator and target of autophagy. Front Oncol.

[26] Cecconi F (2020). Autophagy, replication stress and DNA synthesis, an intricate relationship. Cell Death Differ.

[27] Klionsky DJ (2020). Autophagy participates in, well, just about everything. Cell Death Differ.

[28] Kong E, Kim HD, Kim J (2020). Deleting key autophagy elongation proteins induces acquirement of tumor-associated phenotypes via ISG15. Cell Death Differ.

[29] Alvarado-Ortiz E, de la Cruz-Lopez KG, Becerril-Rico J, Sarabia-Sanchez MA, Ortiz-Sanchez E, Garcia-Carranca A (2020). Mutant p53 gain-of-function: role in cancer development, progression, and therapeutic approaches. Front Cell Dev Biol.

[30] Jacobs SBR, Van Nostrand JL, Bowen ME, Baker JC, Attardi LD (2020). Siva plays a critical role in mouse embryonic development. Cell Death Differ.

[31] Mello SS, Attardi LD (2018). Deciphering p53 signaling in tumor suppression. Curr Opin Cell Biol.

[32] Bieging-Rolett KT, Kaiser AM, Morgens DW, Boutelle AM, Seoane JA, Van Nostrand EL, Zhu C, Houlihan SL, Mello SS, Yee BA (2020). Zmat3 is a key splicing regulator in the p53 tumor suppression program. Mol Cell.

[33] Valente LJ, Tarangelo A, Li AM, Naciri M, Raj N, Boutelle AM, Li Y, Mello SS, Bieging-Rolett K, DeBerardinis RJ (2020). p53 deficiency triggers dysregulation of diverse cellular processes in physiological oxygen. J Cell Biol.

[34] Hayashi A, Hong J, Iacobuzio-Donahue CA (2021). The pancreatic cancer genome revisited. Nat Rev Gastroenterol Hepatol.

[35] Bailey P, Chang DK, Nones K, Johns AL, Patch AM, Gingras MC, Miller DK, Christ AN, Bruxner TJ, Quinn MC (2016). Genomic analyses identify molecular subtypes of pancreatic cancer. Nature.

[36] Yachida S, Iacobuzio-Donahue CA (2013). Evolution and dynamics of pancreatic cancer progression. Oncogene.

[37] Wong C, Tang LH, Davidson C, Vosburgh E, Chen W, Foran DJ, Notterman DA, Levine AJ, Xu EY (2020). Two well-differentiated pancreatic neuroendocrine tumor mouse models. Cell Death Differ.

[38] Beatty GL, Werba G, Lyssiotis CA, Simeone DM (2021). The biological underpinnings of therapeutic resistance in pancreatic cancer. Genes Dev.

[39] Li X, Lee Y, Kang Y, Dai B, Perez MR, Pratt M, Koay EJ, Kim M, Brekken RA, Fleming JB (2019). Hypoxia-induced autophagy of stellate cells inhibits expression and secretion of lumican into microenvironment of pancreatic ductal adenocarcinoma. Cell Death Differ.

[40] Tan X, Sivakumar S, Bednarsch J, Wiltberger G, Kather JN, Niehues J, de Vos-Geelen J, Valkenburg-van Iersel L, Kintsler S, Roeth A (2021). Nerve fibers in the tumor microenvironment in neurotropic cancer-pancreatic cancer and cholangiocarcinoma. Oncogene.

[41] Ito Y, Takeda T, Wakasa K, Tsujimoto M, Sakon M, Matsuura N (2001). Expression of p73 and p63 proteins in pancreatic adenocarcinoma: p73 overexpression is inversely correlated with biological aggressiveness. Int J Mol Med.

[42] Vikhreva P, Melino G, Amelio I (2018). p73 alternative splicing: exploring a biological role for the C-terminal isoforms. J Mol Biol.

[43] Scian MJ, Carchman EH, Mohanraj L, Stagliano KE, Anderson MA, Deb D, Crane BM, Kiyono T, Windle B, Deb SP (2008). Wild-type p53 and p73 negatively regulate expression of proliferation related genes. Oncogene.

[44] Weissmueller S, Manchado E, Saborowski M, Morris JP, Wagenblast E, Davis CA, Moon SH, Pfister NT, Tschaharganeh DF, Kitzing T (2014). Mutant p53 drives pancreatic cancer metastasis through cell-autonomous PDGF receptor beta signaling. Cell.

[45] Riege K, Kretzmer H, Sahm A, McDade SS, Hoffmann S, Fischer M (2020). Dissecting the DNA binding landscape and gene regulatory network of p63 and p53. Elife.

[46] Bellomaria A, Barbato G, Melino G, Paci M, Melino S (2012). Recognition mechanism of p63 by the E3 ligase Itch: novel strategy in the study and inhibition of this interaction. Cell Cycle.

[47] Bellomaria A, Barbato G, Melino G, Paci M, Melino S (2010). Recognition of p63 by the E3 ligase ITCH: effect of an ectodermal dysplasia mutant. Cell Cycle.

[48] Lena AM, Cipollone R, Amelio I, Catani MV, Ramadan S, Browne G, Melino G, Candi E (2010). Skn-1a/Oct-11 and DeltaNp63alpha exert antagonizing effects on human keratin expression. Biochem Biophys Res Commun.

[49] Andricovich J, Perkail S, Kai Y, Casasanta N, Peng W, Tzatsos A (2018). Loss of KDM6A activates super-enhancers to induce gender-specific squamous-like pancreatic cancer and confers sensitivity to BET inhibitors. Cancer Cell.

[50] Tonon G, Wong KK, Maulik G, Brennan C, Feng B, Zhang Y, Khatry DB, Protopopov A, You MJ, Aguirre AJ (2005). High-resolution genomic profiles of human lung cancer. Proc Natl Acad Sci USA.

[51] Adorno M, Cordenonsi M, Montagner M, Dupont S, Wong C, Hann B, Solari A, Bobisse S, Rondina MB, Guzzardo V (2009). A Mutant-p53/Smad complex opposes p63 to empower TGFbeta-induced metastasis. Cell.

[52] Muller PA, Caswell PT, Doyle B, Iwanicki MP, Tan EH, Karim S, Lukashchuk N, Gillespie DA, Ludwig RL, Gosselin P (2009). Mutant p53 drives invasion by promoting integrin recycling. Cell.

[53] Melino S, Nepravishta R, Bellomaria A, Di Marco S, Paci M (2009). Nucleic acid binding of the RTN1-C C-terminal region: toward the functional role of a reticulon protein. Biochemistry.

[54] Nepravishta R, Sabelli R, Iorio E, Micheli L, Paci M, Melino S (2012). Oxidative species and S-glutathionyl conjugates in the apoptosis induction by allyl thiosulfate. FEBS J.

[55] Cabras T, Patamia M, Melino S, Inzitari R, Messana I, Castagnola M, Petruzzelli R (2007). Pro-oxidant activity of histatin 5 related Cu(II)-model peptide probed by mass spectrometry. Biochem Biophys Res Commun.

[56] Mauretti A, Neri A, Kossover O, Seliktar D, Nardo PD, Melino S (2016). Design of a novel composite H2 S-releasing hydrogel for cardiac tissue repair. Macromol Biosci.

[57] Amelio I, Bertolo R, Bove P, Candi E, Chiocchi M, Cipriani C, Di Daniele N, Ganini C, Juhl H, Mauriello A (2020). Cancer predictive studies. Biol Direct.

[58] Consortium ITP-CAoWG (2020). Pan-cancer analysis of whole genomes. Nature.

[59] Liu L, Wang G, Wang L, Yu C, Li M, Song S, Hao L, Ma L, Zhang Z (2020). Computational identification and characterization of glioma candidate biomarkers through multi-omics integrative profiling. Biol Direct.

[60] Chen JC, Tyler AD (2020). Systematic evaluation of supervised machine learning for sample origin prediction using metagenomic sequencing data. Biol Direct.

[61] Larmuseau M, Verbeke LPC, Marchal K (2019). Associating expression and genomic data using co-occurrence measures. Biol Direct.

[62] Mihaylov I, Kandula M, Krachunov M, Vassilev D (2019). A novel framework for horizontal and vertical data integration in cancer studies with application to survival time prediction models. Biol Direct.

[63] Chierici M, Francescatto M, Bussola N, Jurman G, Furlanello C (2020). Predictability of drug-induced liver injury by machine learning. Biol Direct.

[64] Ding L, Bailey MH, Porta-Pardo E, Thorsson V, Colaprico A, Bertrand D, Gibbs DL, Weerasinghe A, Huang KL, Tokheim C (2018). Perspective on oncogenic processes at the end of the beginning of cancer genomics. Cell.

[65] Liang J, Niu Z, Zhang B, Yu X, Zheng Y, Wang C, Ren H, Wang M, Ruan B, Qin H (2021). p53-dependent elimination of aneuploid mitotic offspring by entosis. Cell Death Differ.

[66] Rizzotto D, Villunger A (2021). P53 clears aneuploid cells by entosis. Cell Death Differ.

[67] Khalil A, Jameson MJ (2019). Downregulation of IGF1R expression inhibits growth and enhances cisplatin sensitivity of head and neck squamous cell carcinoma cells in vitro. Horm Cancer.

[68] Amelio I, Mancini M, Petrova V, Cairns RA, Vikhreva P, Nicolai S, Marini A, Antonov AA, Le Quesne J, Baena Acevedo JD (2018). p53 mutants cooperate with HIF-1 in transcriptional regulation of extracellular matrix components to promote tumor progression. Proc Natl Acad Sci USA.

[69] Pitolli C, Wang Y, Mancini M, Shi Y, Melino G, Amelio I (2019). Do mutations turn p53 into an oncogene?. Int J Mol Sci.

[70] Amelio I, Melino G (2015). The p53 family and the hypoxia-inducible factors (HIFs): determinants of cancer progression. Trends Biochem Sci.

[71] Bonnard C, Navaratnam N, Ghosh K, Chan PW, Tan TT, Pomp O, Ng AYJ, Tohari S, Changede R, Carling D (2020). A loss-of-function NUAK2 mutation in humans causes anencephaly due to impaired Hippo-YAP signaling. J Exp Med.

[72] Yuan WC, Pepe-Mooney B, Galli GG, Dill MT, Huang HT, Hao M, Wang Y, Liang H, Calogero RA, Camargo FD (2018). NUAK2 is a critical YAP target in liver cancer. Nat Commun.

[73] Gill MK, Christova T, Zhang YY, Gregorieff A, Zhang L, Narimatsu M, Song S, Xiong S, Couzens AL, Tong J (2018). A feed forward loop enforces YAP/TAZ signaling during tumorigenesis. Nat Commun.

[74] Czirjak G, Enyedi P (2006). Targeting of calcineurin to an NFAT-like docking site is required for the calcium-dependent activation of the background K+ channel. TRESK J Biol Chem.

[75] Decker EL, Nehmann N, Kampen E, Eibel H, Zipfel PF, Skerka C (2003). Early growth response proteins (EGR) and nuclear factors of activated T cells (NFAT) form heterodimers and regulate proinflammatory cytokine gene expression. Nucleic Acids Res.

[76] Lean J, Kirstein B, Urry Z, Chambers T, Fuller K (2004). Thioredoxin-1 mediates osteoclast stimulation by reactive oxygen species. Biochem Biophys Res Commun.

[77] Namiki S, Tomida T, Tanabe M, Iino M, Hirose K (2003). Intracellular delivery of glutathione S-transferase into mammalian cells. Biochem Biophys Res Commun.

[78] Aceto A, Dragani B, Melino S, Allocati N, Masulli M, Di Ilio C, Petruzzelli R (1997). Identification of an N-capping box that affects the alpha 6-helix propensity in glutathione S-transferase superfamily proteins: a role for an invariant aspartic residue. Biochem J.

[79] Angelucci S, Sacchetta P, Moio P, Melino S, Petruzzelli R, Gervasi P, Di Ilio C (2000). Purification and characterization of glutathione transferases from the sea bass (*Dicentrarchus labrax*) liver. Arch Biochem Biophys.

[80] Niitsu H, Hinoi T, Kawaguchi Y, Sentani K, Yuge R, Kitadai Y, Sotomaru Y, Adachi T, Saito Y, Miguchi M (2016). KRAS mutation leads to decreased expression of regulator of calcineurin 2, resulting in tumor proliferation in colorectal cancer. Oncogenesis.

[81] Butera A, Melino G, Amelio I (2021). Epigenetic "drivers" of cancer. J Mol Biol.

[82] Han Y, Ye X, Wang C, Liu Y, Zhang S, Feng W, Huang K, Zhang J (2019). Integration of molecular features with clinical information for predicting outcomes for neuroblastoma patients. Biol Direct.

[83] Kim SY, Jeong HH, Kim J, Moon JH, Sohn KA (2019). Robust pathway-based multi-omics data integration using directed random walks for survival prediction in multiple cancer studies. Biol Direct.

[84] Chowdhury S, Beitel LK, Lumbroso R, Purisima EO, Paliouras M, Trifiro M (2019). A targeted bivalent androgen receptor binding compound for prostate cancer therapy. Horm Cancer.

[85] Bazarbashi S, Su WP, Wong SW, Singarachari RA, Rawal S, Volkova MI, Bastos DA. A narrative review of implementing precision oncology in metastatic castration-resistant prostate cancer in emerging countries. Oncol Ther. 2021. 10.1007/s40487-021-00160-6 PMC8593077 34236692

[86] Oktay K, Santaliz-Casiano A, Patel M, Marino N, Storniolo AMV, Torun H, Acar B, Madak Erdogan Z (2020). A computational statistics approach to evaluate blood biomarkers for breast cancer risk stratification. Horm Cancer.

